# Editorial Chit-Chat

**Published:** 1878-01

**Authors:** 


### Editorial Chit-Chat.

A NEW YEAR.—Your subscription has expired, —blank enclosed,—pay up.

A POINTER.—H, in hunting for wood-cock some time ago, encountered a cow in the willow brush, and utilized her to flush his birds, as he drove her before him. His experience was that, after the first shot, "she put them up too wild."

SECOND MARRIAGES.—A visit to Woodlawn today, brought us in communication with the keeper, who gave us this bit of information: "I can always tell when a widower is about to contract another marriage. When he gives orders to have his wife's grave fixed up, that means he is visiting a lady with a view to marriage. When a tomb stone is erected over his wife's grave, that is a sure indication that he will be married within the month."

FRITZ.—His most recent inspiration—for they are numerous and striking— was when he ran into the room where his mother was seated, and, holding up a lithograph of Lorillard's 5 c. plug tobacco, which represents the Goddess of Liberty, (or some other equally important woman) standing upon a globe, holding a large plug of tobacco in both hands, over her head, exclaimed, "O, Ma! See here! Here's an angel, going up to heaven with her coffin!"

ONE HANDED WOMEN.—Since the introduction of the long street dress with which the ladies now adorn and burden their bodies, we have the somewhat comical appearance of an array of one-handed women upon our promenades. Our women, poor creatures, are forced to devote one hand entirely to carrying their skirts, leaving but one free to be used for the ordinary purposes of life. No opportunity is now afforded the polite gentleman to stop his lady friend upon the street and go through the ceremony of hand-shaking—it can't be done, for the poor woman must needs hold her dress out of the mud with one hand, grasps her purse with the other, and what in canopy she will do when parasol-carrying-time comes again, remains to be seen.

THE INDEX.—With this, the last number of volume thirteen, we give a very full index, so that the numbers can be bound into a volume of 330 pages, giving a book of great value to the physician and the house-wife. Just take a look over the subjects treated upon, and you will be at once struck with the value of the bound volume as a book of reference. We know of no medical journal published that is of as much value to the general practitioner of medicine. Almost every disease is treated of, and the latest and most approved remedies recommended as prescribed by the most celebrated physicians in the world.

The BISTOURY for the past three years, bound and indexed in one volume, will be sent to any address, post paid, upon receipt of $2.50.

THE FLORISTS.—A more kind and lovable people can nowhere be found than the florists. Whether the constant mingling with the flowers softens their tempers and renders amiable their dispositions, we know not, but certain it is that, they are a peculiarly graceful people. In all our interviews with these folks, and they have been not a few, we have never encountered a disobliging or fretful person. Like the flowers they cultivate and handle so tenderly, they seem always to be lovely and agreeable.

A LOANED NEWSPAPER.—On the train, an urbane, well-clad, intelligent looking man, whom we thought to be a gentleman, borrowed our unread newspaper "just for a moment," then comfortably seated himself by the car window and proceeded to enlighten himself upon the transactions of the day, and so interested did he become that the journal did not reach us again until darkness rendered reading impossible to that polite borrower. That fellow was an ass! No, a salamander—an ass would have known better.

A CABINET OF BRAINS.—We spent a day, recently, full of entertainment and information, at Cornell University, with Dr. Wilder, Professor of Comparative Anatomy and Zoology. The Dr. is not only an accomplished lecturer upon his chosen specialty, but an industrious and diligent worker withal, as his very large collection of embryos and brains will attest. Dissections, the most unique and delicate may here be found, requiring a skill in their preparation only obtainable after long practice and study. Brains are shown, of the smallest embryo to the largest, mature, mammal; exposing the sinuses, convolutions, ganglions, origin of nerves, etc., to inspect which, with any degree of carefulness or understanding, would require months of time and patience. We believe this to be the largest and most valuable collection of brain dissections in the world, a visitation to which will repay the scientific man.

NEW MUSICAL INSTRUMENT.—Upon a recent visit to Ithaca, we were shown a new device for supplying music, though the player has no skill as a performer. The instrument is a melodeon, invented by Mr. Horton, played with a treadle, by means of which, and a cog wheel, the music, (consisting of perforations in paper, of more or less length, depending upon the length of the musical tone required), is passed over the reeds, and the harmony is at once afforded. The beauty of this instrument is, it requires no skill to play it. Any-one who can work the treadle and place the sheet of music in position, can furnish the tune.

---

POOR JIM.—He is no more, has departed, shuffled off this mortal coil, passed in his checks and died. Died, did he, because he knew not enough to come in out of the rain, but persistently sat upon his favorite perch, through a drenching rain, "caught his death-of-cold," and went off in a galloping consumption.—Poor Jim, we sorrow for him, as we look from our sanctum window and see his vacant perch, where, three short months ago he afforded us amusement and an article for our journal: —Jim was a versatile, knowing creature, and we doubt not, ere this, if his qualities are properly appreciated, that he has become a black faced babboon, or perhaps a negro baby, according to the Darwinian theory. But wherever Jim may be located or whatever his physical metamorphosis, we wish him a genuine success. We still cling to his tail feathers, keeping them in memory of the jolliest and funniest of Crows.

---

SPIRAL SHIRT STUDS.—The chap that invented them deserves to have his shirt stuck full of them, front and rear. Should have the caudal appendage of said garment bristling with them, and then be forced to sit and listen to Talmage's lecture on Happy Homes, for the balance of the season. Was there ever a more barbarous instrument contrived for the torture of lean mankind ? We will answer that conundrum ourself—No! there never was! Every time you inflate your lungs, the scratching point of that spiral wire penetrates your flannel and imprints an unknown hieroglyphic upon your sternum. To hug a pretty girl—your daughter, is impossible, if you have any desire to perform the ceremony heartily; while, to salute your wife, if she be fat, is one of the impossibilities, without bending yourself in the form of a letter C, and resisting all demonstrations looking toward an embrace, that those spiral darts may not be thrust into your quivering flesh. We want no more presents of spiral shirt studs, even if they be set with diamonds, until the present punctures in our chest are healed and the memory of them obliterated.

---

STEALING A CAMEL.—We have heard somewhere, of a chap who stole a saw-log, returned the following night to capture the mill, and was caught at it; but the incident we are about to relate is the only one upon record, we imagine, where so bulky a theft was perpetrated as that of confiscating a camel.

At one of the colleges in this State, not many years ago, the news was conveyed to the Professor of Zoology, that a camel—a real live camel, was feeding upon the shores of the lake. The field glass was at once brought upon the grazing animal by the incredulous Professor, and sure enough, there the strange beast, with his characteristic hump, was found to be. At once, a trio of enthusiastic students in natural history, armed with shot guns and rifles started in pursuit of the strange quadruped, determined upon his capture, that another specimen might be added to their already interesting museum. Arriving upon the spot, they were encountered by a man who informed the boys that the camel belonged to the farmer upon whose premises they were, but who was just then in a neighboring town. This was an unpleasant revelation, for, with the boys came also a drayman with cart and horse to convey the game to the college. The conveyor of bulky packages, loath to lose his ten dollar fee, volunteered to seek the owner of the coveted prize, and, if possible, negotiate a sale of the beast to the boys in waiting. The farmer was found, and this was the distressing tale with which he assailed that unsuspecting owner of the innocent beast of a wandering menagerie: "Say, mister, them college fellers were out shootin on yer farm and killed that elephant or rhinoseros or whatever you call 'im, and a nother feller said he'd give ten dollars for his hide to stuff—you'd better take it, for a ded critter's no use to you!" The farmer contemplated the situation for a moment, concluded ten dollars was better than a carcass of a camel, and gave the diplomat a bill of sale of the creature. With this the cartman returned to find that the boys had given the camel chloroform, had him loaded upon the cart, and were making off with him to the classic precincts of the college.

The skin and bones of the poor camel, mounted, now adorn the college museum, and we thought we detected a peculiar curl of the creature's nostrils, as though he had not yet forgotten the sickening odor of that cap full of chloroform.

